# Pain assessment scales in newborns: integrative review

**DOI:** 10.1016/j.rpped.2014.04.007

**Published:** 2014-12

**Authors:** Gleicia Martins de Melo, Ana Luíza Paula de Aguiar Lélis, Alline Falconieri de Moura, Maria Vera Lúcia Moreira Leitão Cardoso, Viviane Martins da Silva

**Affiliations:** Universidade Federal do Ceará (UFC), Fortaleza, CE, Brazil

**Keywords:** Pain measurement, Newborn, Pain

## Abstract

**OBJECTIVE:**

To analyze studies on methods used to assess pain in newborns.

**DATA SOURCES:**

Integrative review study of articles published from 2001 to 2012, carried out in the following databases: Scopus, PubMed, CINAHL, LILACS and Cochrane. The sample consisted of 13 articles with level of evidence 5.

**DATA SYNTHESIS:**

29 pain assessment scales in newborns, including 13 one-dimensional and 16 multidimensional, that assess acute and prolonged pain in preterm and full-term infants were available in scientific publications.

**CONCLUSION:**

Based on the characteristics of scales, one cannot choose a single one as the most appropriate scale, as this choice will depend on gestational age, type of painful stimulus and the environment in which the infant is inserted. It is suggested the use of multidimensional or one-dimensional scales; however, they must be reliable and validated.

## Introduction

The interest in studies on pain and its assessment tools is on the rise, due to the subjectivity inherent to its measurement, especially in children, which, considering the characteristics of the age group or developmental delays, fail to report or properly indicate the painful event. Considering these aspects, it is recommended the use of validated tools for pain assessment in children, especially critically ill ones.^1^

The methods used for the assessment of painful events can be divided into three categories: measurement of physiological responses of pain, observations of behaviors related to pain, and verbal or written descriptions of pain and/or associated variables. There are measures of pain intensity (one-dimensional) and measures of multiple dimensions of pain (multidimensional).^2^

The one-dimensional tools are designed to measure the presence or absence of pain and have been frequently used in hospitals and/or clinics to obtain fast, noninvasive, valid information on pain and analgesia. As for the multidimensional tools, they are used to assess sensory, affective and evaluative components that are reflected in the language used to describe the painful experience.^3^

Although no specific pain scale has demonstrated its superiority as a reliable biomarker and gold standard yet,^4^ it is noteworthy that, for some authors, the use of multidimensional scales in newborns (NBs) is the most adequate, as they assess behavioral responses associated with physiological responses to pain, making the approach as comprehensive as possible, considering that the reporting of pain cannot be expressed by this population.^5^^,^ 6

Among the several multidimensional pain scales for children and infants, the most studied are the Neonatal Facial Coding System (NFCS), the Neonatal Infant Pain Scale (NIPS) and the Premature Infant Pain Profile (PIPP).^7^

Moreover, when evaluating pain, the health professional is influenced by aspects related to professional experience, measurement methods that are easy to use, type of pain, observed signs of pain, the child's age, type of painful procedure, clinical status, psychometric properties, interpretation criteria, methods that are easy to apply, and experience of having used it in other health services.^8^^-^ 10

It is believed that the scarcity of teaching strategies and discussion about pain during the undergraduate, residency and post-graduate courses in the health care area, as well as in daily clinical practice are factors that influence the difficulty of evaluating pain in the neonatal period,^11^^,^ 12 in addition to the variety of tools and the specificity of their characteristics, associated with the lack of knowledge on the variation of their applicability for pain assessment in children. Thus, the identification and characteristics of pain measurement tools published in studies in the pediatric and neonatal areas can offer health professionals a practical means to choose the most appropriate one for their area of activity, aiding in clinical decision-making.

Given this context, we aimed to search the databases of scientific literature for studies related to tools used for pain assessment in newborns. Thus, the following questions were raised: What are the tools used to assess pain in newborns? What are the main characteristics of each tool and its applicability in neonatology? The answers to these questions will help to present the evidence on the subject. Therefore, our objective was to analyze, in scientific articles, the methods used to assess pain in newborns.

## Method

This is an integrative review, due to the convenience of analysis of the literature regarding the completed studies, to identify tools used for pain assessment in newborns.

The review followed these steps: establishing the guiding question of the study, formulation of inclusion and exclusion criteria, definition of information to be extracted from selected studies, assessment of studies included in the integrative review, interpretation of results, presentation of the review and synthesis of knowledge.^13^

The following questions were raised to meet the research objectives: What are the tools used to assess pain in newborns? What are the main psychometric properties evaluated and what is their applicability in neonatology?

Inclusion criteria were defined as: study available electronically in the selected databases in Portuguese, English and Spanish; which analyzed the psychometric characteristics of tools used for pain assessment in newborns, and that were published from 2001 to 2012. Editorials, letters to the Editor, reflective studies, case reports, annals of scientific events (abstracts) and duplicate publications were excluded.

The bibliographic survey was carried out in October and November of 2012 by two researchers separately, who searched five databases, according to the following sequence: Latin American Literature in Health Sciences (LILACS), Cumulative Index to Nursing and Allied Health Literature (CINAHL), Cochrane, SCOPUS and PubMed. It is worth mentioning that the search in the databases ended on 15 November 2012.

The controlled descriptors "*medição da dor*" and "*recém-nascido*", found in the Health Sciences Descriptors (DECS) were used for the search in the LILACS database, whereas for the other databases, the terms "pain measurement" and "newborn", found in the Medical Subject Headings (MESH) were used. The same sequence was followed in the insertion of descriptors for searches in the five chosen databases, and as a search cutoff it was established that articles published in the last eleven years would be selected, in order to include the largest number of publications on the study topic.

After the selection process and the identification of articles that followed the established inclusion criteria, we identified the following articles: none in the LILACS database in national journals; five in CINAHL; none in Cochrane; four in SCOPUS, and eight in PubMed, in international journals. After excluding the duplicate studies published in more than one database, we had five in CINAHL, four in SCOPUS and six in PubMed - a total of 15 studies. Of these, two were excluded, as they aimed to discuss scales for pain assessment in children aged three years and older, even though they were initially selected for exhibiting the same scales used to assess pain in both infants and children. Thus, 13 studies comprised the final sample of this review. 

To define the information extracted from the selected studies, a three-part instrument was developed. The first, related to the identification of articles with the items: title of the study and the journal, country, language, year of publication and authors' names. The second, related to the methodological characteristics of the articles, containing: type of publication, study objective or question, population and sample, child's age, gender, sample number, location, person responsible for applying the tool, clinical conditions of the newborn, use of another tool in the study; and the third part, related to the tool data, such as type, name and abbreviation of the tool, type of pain, time of application and psychometric data. 

Study titles were read in the selection phase, followed by the summaries or abstracts. The articles were then read and analyzed in full, including data related to the measurement of pain in infants. The results were shown in tables, and the discussion was based on literature relevant to the subject.

The studies were classified according to the level of evidence: Level I - evidence from systematic review or meta-analysis of randomized controlled clinical trials or from clinical guidelines based on systematic reviews of randomized controlled trials; Level II - evidence derived from at least one randomized, controlled, well-designed trial; Level III - evidence obtained from well-designed clinical trials without randomization; Level IV - evidence from well-designed cohort and case-control studies; Level V - evidence from systematic review of descriptive or quantitative studies; Level VI - evidence based on the opinion of experts and/or expert committee reports.^14^

## Results and discussion

### Characterization of the selected studies 

The majority of articles, 12 (92.3%), were written in English. Nine (69.2%) had been published since 2007, nine (69.2%) in medical journals, four (30.67%) in nursing journals, and 10 (76.9%) were validation studies. The predominance of this type of design is justified by the purpose of this study, which aimed to identify tools to measure pain and their assessed psychometric properties. As for the level of evidence, all were level V.^14^

In the 13 articles, we identified 29 validated scales for pain assessment in newborns. Of these, 13 are one-dimensional and are 16 multidimensional scales. The one-dimensional tools use a single indicator of pain assessment: physiological or behavioral, whereas multidimensional tools are those that provide a more comprehensive assessment of pain, as they include both the physiological and behavioral aspects. The most commonly used physiological indicators are vital signs such as heart rate and oxygen saturation, and behavioral measures such as facial expression, crying and motor activity.^15^

Most selected articles classify the scales according to the type of pain, as Acute and Prolonged/Chronic pain. This classification considers that acute pain is frequently caused by nociceptive stimuli resulting from tissue lesions caused by procedures or accidental lesions, and it usually disappears as wound healing occurs. In cases of chronic or prolonged pain, an inflammatory process often occurs, triggered by or as the aftermath of an acute painful phenomenon.^16^

The tools identified are shown in tables 1 and 2 and classified as one-dimensional and multidimensional, as well as regarding the type of pain, as acute and prolonged.

**Table 1:**
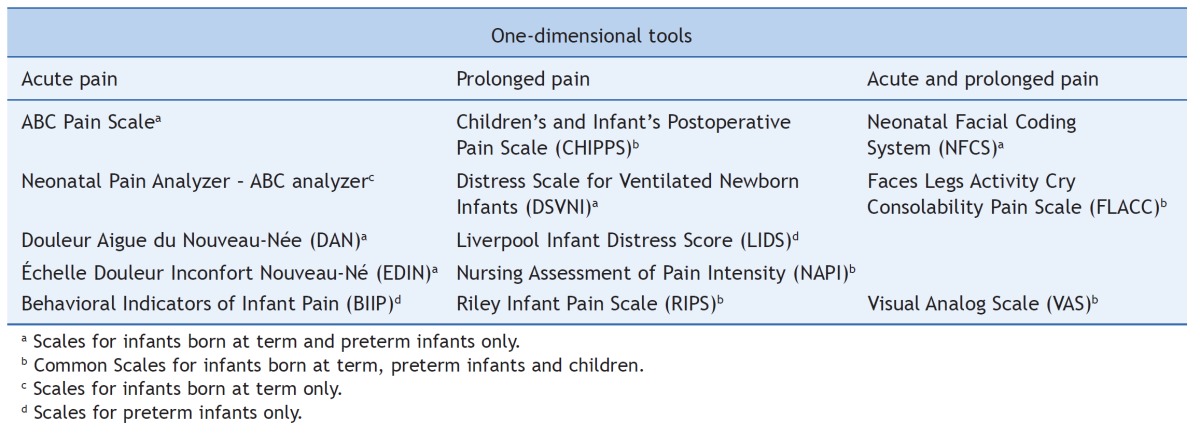
Distribution of tools according to one-dimensional classification and types of pain. Fortaleza, Brazil, 2012.

**Table 2:**
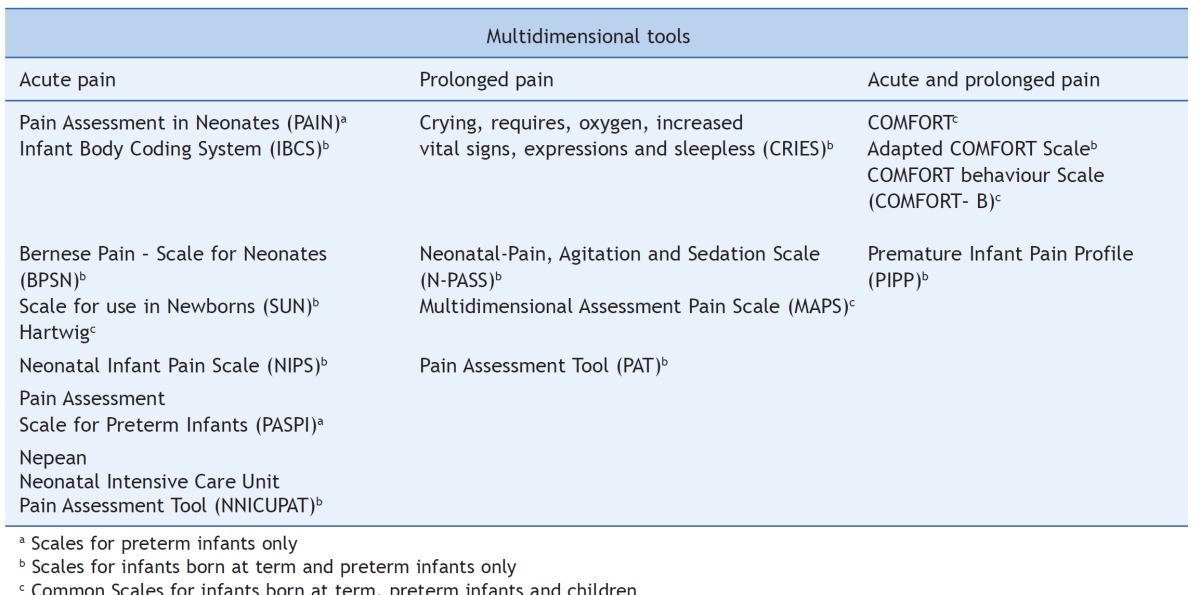
Distribution of tools according to a multidimensional classification and types of pain. Fortaleza, Brazil, 2012.

Most tools are one-dimensional, for acute pain, and use some of the following behavioral indicators: facial expression, crying and motor activity. In the case of the ABC pain scale, for instance, it assesses pain by the crying characteristics of the newborn: timbre, rhythm of crying bouts and constancy of crying intensity. Meanwhile, the Neonatal Pain Analyzer - ABC analyzer uses other aspects from the former indicator, in addition to timbre, such as the normalized root mean square (RMS) amplitude and the presence of a characteristic frequency- and amplitude-modulated crying feature, defined as "siren cry".^17^^-^ 19

Crying is the most primitive form of communication, and is considered a sign, a symptom and an indicator. The meaning of crying is still unclear, as its different characteristics can refer to different levels of stress related to several causes, such as pain, hunger or discomfort.^20^^-^ 21

Also for acute pain, we mention the DAN, EDIN and BIIP scales. In addition to the behavioral indicator related to facial expression, the authors also used other behavioral aspects, namely: DAN, with facial expression, limb movements, and vocal expression; EDIN, through facial expression, body movements, sleep quality, quality of interaction, comfort/consolability; and BIIP, with sleep/wake state, five different facial grimaces and two different hand movements.^17^^,^ 22 ^-^ 23

Of the one-dimensional scales for prolonged pain, VAS evaluates only the facial expression,24 and LIDS assesses body movements, excitability, flexion of the fingers and the first toe, muscle tone, facial expression (quantity and quality) and sleep. CHIPPS, RIPS and NAPI associated the three following behavioral indicators: facial expression, crying and motor activity, as well as others, such as response to touch in NAPI, consolability and response to touch.^17^^,^ 25 ^-^ 27

The DSVNI scale used behavioral indicators based on five scales - NFCS, The Infant Body Coding System (IBCS), Neonatal Behavioral Assessment Scale, Assessment of Preterm Infants' Behavior and Gustave Roussy Child Pain Scale, which were not disclosed in the selected study because this scale has not been published and is not being used in clinical practice.^17^^,^ 28

The NFCS and the FLACC scales evaluated acute and prolonged pain. The NFCS uses only aspects of facial expression such as forehead and squinted eyes, deepening of the nasolabial furrow and horizontal mouth stretch, while FLACC measures pain using all types of behavioral indicators: facial expression, lower limb movements, bodily activity, crying and difficulty in consoling the infant.^17^^,^ 29 ^-^ 31

In newborns and infants, pain tends to manifest through crying and body movements, facial expressions or even apathy. In children aged between one and three years, crying may arise accompanied by verbalizations or gestures located in the region of the pain focus, and by certain restless, violent or tantrum movements.^32^

As crying, facial expressions and motor activity are indicators observed in other situations experienced by the newborn, such as stress and discomfort, for instance, some researchers have sought to develop scales that associated behavioral and physiological indicators to obtain more accurate pain assessment.

The multidimensional expressions of pain indicate that the assessment should not focus only on the selected behavioral signs, but also capture all possible expressions of pain.33 Responses to pain are also influenced by stimulus type, sleep-wake state, developmental age, disease severity, use of pharmacological agents and their amount, type and time of exposure of pain.^34^

Of the multidimensional tools for acute pain assessment, it is worth mentioning PAIN, with indicators such as facial expression, crying, breathing pattern, movement of extremities, state of alertness, oxygen saturation and heart rate;^35^ the IBCS scale, through facial expression, body movements, characteristics of crying, heart rate;^36^ and the BPSN scale, which uses state of alertness, crying duration, time to calm down, skin color, facial expression, posture, breathing pattern, heart rate and oxygen saturation.^37^

The SUN scale has indicators of heart rate, breathing, mean arterial pressure, state of alertness, movement, muscle tone, and facial expression.^38^ The NIPS scale has facial expression, crying, breathing patterns, upper limb movements, lower limb movements and state of alertness,^17^^,^ 25 and the Hartwig scale assesses motor response, facial expression, eye opening, respiratory rate and reaction to orotracheal aspiration.^39^

The PASPI scale uses the transition between the sleep-wake states, facial expressions, changes in heart rate and oxygen saturation, body and limb movements and behavior of the hands.^40^ The NNICUPAT scale works through facial expression, body movements, skin color, oxygen saturation, respiratory rate, heart rate and pain perception by the nurse.^17^^,^ 41

To evaluate prolonged pain, the CRIES scale works with indicators such as crying, facial expression, oxygen saturation, vital signs and sleep patterns.^25^ The N-PASS scale works with crying/irritability, status/behavior, facial expression, muscle tone/extremities, vital signs (heart rate, respiratory rate, blood pressure and/or oxygen saturation).^42^ The MAPS scale uses heart rate and blood pressure, breathing patterns, facial expression, body movements and state of alertness,^43^ and the PAT scale evaluates facial expressions, crying, posture, sleep, perception of the nurse, skin color, heart rate, breathing pattern, blood pressure and oxygen saturation.^17^^,^ 44 ^-^ 45

The COMFORT scale is a multidimensional tool for pain assessment that uses behavioral indicators: state of alertness, agitation, respiratory reaction, crying, general movements, muscle tone and facial expression, and physiological ones: blood pressure and heart rate. This scale was specifically developed to assess measures of distress caused by pain in children aged 0-18 years old, admitted to the ICU.^17^^,^ 46 ^-^ 47

The Adapted COMFORT scale originates from the COMFORT scale, which was submitted to a validation study with preterm infants with less than 35 weeks of gestational age, using all items of the original scale, except the evaluation of invasive blood pressure.^17^^,^ 46 ^-^ 47 The COMFORT-B was derived from the original COMFORT scale, excluding the two physiological parameters (heart rate and mean arterial blood pressure), keeping only behavioral indicators: state of alertness, agitation, respiratory reaction, crying, general movements, muscle tone and facial expression. However, the respiratory reaction aspect, considered a physiological component, maintains this scale as a multidimensional one.^17^^,^ 46 ^-^ 48

The PIPP scale assesses gestational age, state of alertness, heart rate, oxygen saturation and facial expression (frowning, closed eyes, deepening of the nasolabial furrow). It is the only multidimensional scale that, among its indicators, includes gestational age to evaluate pain in full-term and preterm newborns.^17^^,^ 49

As for the age range by scale, it was observed that the CHIPPS and COMFORT-B scales assess pain in children aged zero to five years; the FLACC, RIPS, COMFORT and NAPI scales, from zero to three years; VAS, from zero to 4 years; MAPS, from zero to 31 months, and the Hartwig scale, from zero to one year,^17^^,^ 39 ^,^ 44 ^,^ 50 which are common to newborns and children. 

Regarding the painful stimulus, the scale use was observed in calcaneal puncture (ABC pain scale, Neonatal Pain Analyzer - ABC analyzer, DAN, IBCS, PIPP, NIPS, PASPI), in venipuncture (DAN, BIPP, PIPP, NIPS), in mechanical ventilation (EDIN, N-PAN, NNICUPAT), in orotracheal aspiration (PAIN, COMFORT), after surgery (EDIN, CHIPPS, FLACC, LIDS, NAPI, RIPS, VAS, NFCS, N-PAN, PIPP, PAT, MAPS, COMFORT, COMFORT *Scale*), in painful routine procedures (DSVNI) and in burns (VAS).^17^^,^ 44

When correlating the tools to the application context, it was observed that the VAS scale was evaluated in patients with rheumatoid arthritis,^48^ Hartwig in the newborn under mechanical ventilation during tracheal aspiration,^39^ the COMFORT-B in children with Down syndrome in ICUs^48^ and the BPSN in newborns with and without positive pressure ventilation.^37^ It is noteworthy that the same scale can be used to assess pain in different contexts.^51^

Regarding the psychometric properties of pain scales, criterion validity was predominant in the selected studies. The PASPI^40^ and the COMFORT-B were the only tools that showed content, criterion^47^^,^ 48 and construct validity.^48^^,^ 50

According to the data of the systematic review study, the VAS was compared to the Modified Infant Pain Scale (MIPS) and showed a high degree of agreement when classifying the newborns as comfortable or not comfortable after elective surgery. It was also observed that this scale was used to validate the NIPS, COMFORT and NNICUPAT. Together with the COMFORT scale to validate NFCS and with the PIPP to validate BPSN, and also with the FLACC to validate MAPS in children aged 0-31 months.^17^ Therefore, VAS was one of the scales more often used for validation criterion.

Still on the validation criteria, the COMFORT scale was used in newborns with the VAS scale to validate the NFCS. The reliability of the scale was given by Kappa (0.62-0.84) and by the intraclass coefficient (0.85).^46^ The COMFORT-B scale showed adequate internal consistency when compared to the Numerical Rating Scale (NRS) in children with Down syndromem with Cronbach's alpha value (0.84-0.87).^48^

The interobserver reliability of the NFCS scale was evaluated in several studies, both in the modified version of the scale with four conventional measures and in the conventional version, with ten measures. The version of the scale with ten measures obtained a value of 0.89, with a mixed sample of full-term and preterm newborns, whereas the sample with four measures obtained an interobserver reliability of 0.91.^17^^,^ 52

In a review carried out to assess the measure properties and intervention studies with the PIPP scale, it was observed that the tool remains a reliable and valid measure for the assessment of acute pain in children. The interclass reliability was excellent (>0.89) and the intra-rater reliability was 0.95.^49^ Another review study, which used the same scale of pain in full-term and preterm newborns, achieved an excellent interobserver reliability of 0.93 to 0.96, and an intraobserver reliability of 0.94 to 0.98.^17^

## Conclusion

The present study showed that there are at least 29 available scales that assess pain in newborns in scientific publications in the neonatology area, of which 13 are one-dimensional and 16 multidimensional, which include preterm and full-term newborns in situations of acute and prolonged pain. 

The selected articles showed level V evidence, i.e., evidence originating from systematic reviews of descriptive or quantitative studies, in which most of them were methodological validation studies, consistent with the objectives of the present study.

Based on the knowledge of the characteristics of each scale, we cannot choose the most appropriate one, as the choice will depend on the gestational age, type of painful stimulus and the context in which the newborn is inserted. Therefore, it is noteworthy that, considering the aforementioned studies, there is still no gold standard scale for pain assessment in newborns. The health care professional should use validated, reliable, safe and practical scales at the bedside, which may be one- or multidimensional scales, especially given the divergences found in the literature.

It is emphasized that pain assessment in the neonatal period should be multidisciplinary; due to the subjectivity of the evaluated phenomenon and available scales, when more professionals from different healthcare areas evaluate the same newborn using different scales, perhaps the objectivity of this assessment can be increased. 

We also emphasize the urgent need for services to have, use and update routines and written protocols for the assessment and treatment of pain in newborns, as well as training and qualification of professionals working in these units, ensuring the practical application of knowledge related to prevention, assessment and management of pain, in order to standardize the performance of the service professionals and allow appropriate treatment of the newborns.

Specifically related to pain assessment scales, it is important that, before they are applied, the health care professional know the details of the assessed dimensions, the operationalization of use and the necessary equipment for evaluation consistent with the proposal of the tool. 

We recognize the need to use more specific and accurate methods for neonatal pain assessment, due to the subjectivity of pain, mainly in a population that does not verbalize pain sensation. Thus, the aim is that, through this study, the multidisciplinary team of professionals can choose the most appropriate pain assessment scale for their field of expertise, time availability, population, type of pain and validity.
