# Supplementary material for: Pain assessment scales in newborns: integrative review
Source: Rev Paul Pediatr. 2014 Dec;32(4):395–402. doi: 10.1016/j.rpped.2014.04.007 (PMC4311795; doi:10.1016/j.rpped.2014.04.007)
Supplement: Translation

# Translation: Escalas de avaliação de dor em recém-nascidos: revisão integrativa

**Authors:** Gleicia Martins de Melo, Ana Luíza Paula de Aguiar Lélis, Alline Falconieri de Moura, Maria Vera Lúcia Moreira Leitão Cardoso, Viviane Martins da Silva

**Affiliations:** Universidade Federal do Ceará (UFC), Fortaleza, CE, Brasil

**Keywords:** Medição de dor, Recém-nascido, Dor

## Abstract

**OBJETIVO:**

Analisar, em artigos científicos, os métodos utilizados para avaliação da dor em recém-nascidos.

**FONTES DOS DADOS:**

Estudo de revisão integrativa de artigos publicados de 2001 a 2012, realizado nas bases: Scopus, PubMed, CINAHL, LILACS e Cochrane. A amostra constitui-se de 13 artigos, todos com níveis de evidência 5.

**SÍNTESE DOS DADOS:**

Constataram-se pelo menos 29 escalas de avaliação de dor em recém-nascidos disponíveis em publicações cientificas, sendo 13 unidimensionais e 16 multidimensionais, que contemplam recém-nascidos pré-termo e a termo, para avaliação de dor aguda e prolongada.

**CONCLUSÃO:**

Com base nas características das escalas, não se pode eleger a mais adequada, pois a escolha dependerá da idade gestacional, do tipo de estímulo doloroso e do contexto em que o recém-nascido se apresenta. Sugere-se a utilização de escalas unidimensionais ou multidimensionais, porém, estas devem ser validadas e confiáveis.

## Introdução

O interesse em estudos que abordam a dor e seus instrumentos de avaliação é ascendente, em virtude da subjetividade inerente a sua mensuração, principalmente em crianças, que, devido às características próprias da faixa etária ou aos atrasos de desenvolvimento, não conseguem relatar ou indicar adequadamente o evento doloroso. Em razão desses aspectos, recomenda-se o uso de instrumentos validados para a avaliação da dor de crianças, especialmente, as criticamente doentes.^1^

Os métodos para a avaliação do fenômeno doloroso podem ser divididos em três categorias: medida de respostas fisiológicas da dor, observações de comportamentos relacionados à dor e descrições verbais ou escritas da dor e/ou variáveis associadas. Existem medidas da intensidade da dor (unidimensionais) e medidas das múltiplas dimensões da dor (multidimensionais).^2^

Os instrumentos unidimensionais são designados para quantificar a presença ou ausência da dor e têm sido usados frequentemente em hospitais e/ou clínicas para se obterem informações rápidas, não invasivas, válidas sobre a dor e a analgesia. Já os instrumentos multidimensionais são empregados para avaliar componentes sensoriais, afetivos e avaliativos que estão refletidos na linguagem usada para descrever a experiência dolorosa.^3^

Embora, nenhuma escala específica de dor até o momento demonstrou superioridade, com biomarcador confiável e de padrão ouro,^4^ vale ressaltar que para alguns autores, o uso das escalas multidimensionais em recém-nascidos (RN) são mais adequadas, por avaliarem respostas comportamentais associadas às respostas fisiológicas à dor, tornando a abordagem mais completa possível, tendo em vista que o relato de dor não pode ser manifestado nesse público.^5^^,^ 6 Dentre as várias escalas de dor multidimensionais para crianças e RN, as mais estudadas são o Sistema de Codificação da Atividade Facial (SCAFN), a Escala de Avaliação de Dor (NIPS) e o Perfil de Dor do Prematuro (PIPP).^7^

Ainda, para avaliar a dor, o profissional de saúde é influenciado por aspectos relacionados à experiência profissional, facilidade de uso dos métodos de medição, tipo de dor, sinais de dor apresentados, idade da criança, tipo de procedimento doloroso, situação clínica, propriedades psicométricas, critérios de interpre tação, facilidade de aplicação, expe riência de utilização em outros serviços de saúde.^8^^-^ 10

Acredita-se que a escassez de estratégias de ensino e discussão sobre a dor durante os cursos de graduação, residência, pós-graduação stricto sensu e lato sensu na área da saúde, assim como na prática clínica diária são fatores que influenciam na dificuldade de avaliação da dor no período neonatal,^11^^,^ 12 acrescido da variedade de instrumentos e a especificidade de suas características, associados ao desconhecimento das variações em sua aplicabilidade para avaliação de dor em pediatria. Por isso, a identificação e as características dos instrumentos de medições de dor publicados em pesquisas na área pediátrica e neonatal podem oferecer aos profissionais de saúde um meio prático de consulta para a escolha mais adequada a sua área de atuação, auxiliando a tomada de decisão clínica. 

Em face desse contexto, surgiu a necessidade de buscar, nas bases de dados de literatura científica, as pesquisas relacionadas aos instrumentos de avaliação de dor em RN. Diante disso, surgiram as indagações: Quais são os instrumentos utilizados para avaliação da dor em RN? Quais são as principais características de cada instrumento e sua aplicabilidade em neonatologia? As respostas a essas questões contribuirão para apresentar as evidências sobre a temática. Estabeleceu-se, então, como objetivo, analisar em artigos científicos os métodos utilizados para avaliação da dor em recém-nascidos.

## Metódo

Trata-se de um método de revisão integrativa, em virtude da conveniência de análise da literatura em relação às pesquisas concluídas, para identificar instrumentos de avaliação da dor em RN.

Seguiu-se as etapas: estabe lecimento da questão norteadora do estudo, formulação dos critérios de inclusão e exclusão, definição das infor mações a serem extraídas das pesquisas selecionadas, avaliação dos estudos incluídos na revisão integrativa, interpretação dos resultados, apresentação da revisão e síntese do conhecimento.^13^

Elaborou-se as seguintes questões para atender os objetivos da pesquisa: Quais são os instrumentos utilizados para avaliação da dor em RN? Quais são as principais propriedades psicométricas avaliadas e sua aplicabilidade em neonatologia?

Como critérios de inclusão foram definidos: pesquisas disponíveis eletronicamente nas bases de dados selecionadas nos idiomas português, inglês e espanhol, que tratam sobre as características psicométricas dos instrumentos de avaliação de dor em RN e que foram publicados a partir de 2001 até 2012. Foram excluídos os editoriais, cartas ao Editor, estudos reflexivos, relatos de experiência, anais de eventos científicos (resumos) e publicações duplicadas.

O levantamento bibliográfico ocorreu em outubro e no-vembro de 2012, isoladamente, por duas avaliadoras, quando se efetuou busca a cinco bases de dados, em atenção à seguinte sequência: Literatura Latino-Americana em Ciências de Saúde (LILACS), *Cumulative Index to Nursing and Allied Health Literature* (CINAHL), Cochrane, SCOPUS e Publicações Médicas (PubMed). Ressalta-se que a busca nas bases de dados, encerrou-se no dia 15 de novembro de 2012. 

Utilizaram-se os descritores controlados "medição da dor" e "recém-nascido", constantes nos Descritores em Ciências da Saúde (DECS) para a busca na base de dados LILACS e, para as demais bases de dados, foram utilizados *pain measurement *e* newborn*, constantes no *Medical Subject Headings* (MESH). Obedeceu-se a mesma sequência na inserção dos descritores para as buscas nas cinco bases escolhidas e, como limite de busca, estabeleceu-se que seriam selecionadas as publicações dos últimos onze anos, a fim de abranger o maior quantitativo de publicações a respeito da temática em estudo.

Após o processo de seleção e a identificação dos artigos que obedeceram aos critérios de inclusão estabelecidos, identificaram-se os seguintes artigos: nenhum na base de dados LILACS, em periódico nacional; cinco no CINAHL, nenhum no Cochrane, quatro no SCOPUS e oito no PubMed, em periódicos internacionais. Com a exclusão de pesquisas repetidas em mais de uma base de dados, restaram cinco no CINAHL, quatro no SCOPUS e seis no PubMed, perfazendo um total de 15 estudos, destes dois foram excluídos por terem como objetivo discorrer sobre escalas que avaliam a dor em crianças a partir de três anos, mas que foram inicialmente selecionados por apresentarem as mesmas escalas usadas tanto para avaliar a dor em RN e em crianças. Assim, restaram 13 estudos para a amostra final desta revisão.

Para a definição das informações extraídas das pesquisas incluídas, foi elaborado um instrumento com três partes. A primeira, sobre a identificação dos artigos com os itens: título do estudo e do periódico, país, idioma, ano de publicação e nomes dos autores. A segunda, referente às características metodo lógicas dos artigos, contendo: tipo de publicação, objetivo ou questão de investigação, população e amostra, idade da criança, sexo, número da amostra, local, responsável pela aplicação do instrumento, condições clínicas do RN, uso de outro instrumento na pesquisa e a terceira parte referente aos dados do instrumento, como tipo de instrumento, nome e sigla, tipo de dor, tempo de aplicação e dados psicométricos. 

Na fase de seleção dos estudos incluídos, ocorreu a leitura dos títulos, seguida da leitura do resumo ou *abstract. *Os artigos foram lidos e analisados na íntegra seguindo o roteiro, incluindo dados relacionados à medição da dor em RN. Os resultados foram apresentados em tabelas e a discussão se realizou com base na literatura pertinente ao assunto.

Os estudos foram classificados quanto ao nível de evidência: nível I - evidências provenientes de revisão sistemática ou metanálise de ensaios clínicos randomizados controlados ou oriundos de diretrizes clínicas baseadas em revisões sistemáticas de ensaios clínicos randomizados controlados; nível II - evidências derivadas de, pelo menos, um ensaio clínico randomizado controlado bem delineado; nível III - evidências obtidas de ensaios clínicos bem delineados sem randomização; nível IV - evidências provenientes de estudos de coorte e de caso-controle bem delineados; nível V - evidências originárias de revisão sistemática de estudos descritivos ou quantitativos; nível VI - evidências oriundas de opinião de autoridades e/ou relatório de comitês de especialistas.^14^

## Resultados e discussão

### Caracterização dos estudos selecionados

A maioria dos artigos, 12 (92,3%), encontrava-se no idioma inglês. Nove (69,2%) foram publicados a partir de 2007, nove (69,2%) em revistas científicas médicas, quatro (30,67%) em periódicos de enfermagem, e 10 (76,9%) tratavam-se de estudo de validação. A predominância desse tipo de delineamento se justifica pela proposta deste estudo, que visou identificar instrumentos de medição de dor e suas propriedades psicométricas avaliadas. Segundo o nível de evidência, todos se enquadraram no nível V.^14^

Nos 13 artigos, identificaram-se 29 escalas validadas para a avaliação de dor em RN. Destas, 13 são escalas unidimensionais e 16 multidimensionais. Os instrumentos unidimensionais utilizam um único indicador de avaliação de dor: fisiológicos ou comportamentais. Enquanto que os instrumentos multidimensionais são aqueles que fornecem uma avaliação mais abrangente da dor, visto que contemplam tanto os aspectos fisiológicos como os comportamentais. Os indicadores fisiológicos mais usados são os sinais vitais, como frequência cardíaca e saturação de oxigênio e as medidas comportamentais, a expressão facial, o choro e a atividade motora.^15^

A maioria dos artigos selecionados classifica as escalas segundo o tipo de Dor Aguda e Prolongada/Crônica. Tal classificação considera que a dor aguda frequentemente é causada por estímulos nociceptivos resultantes de lesões teciduais por procedimentos ou acidentais que costuma desaparecer à medida que ocorre cicatrização da ferida. Em casos de dor prolongada ou crônica, frequentemente, ocorre processo inflamatório deflagrado ou em continuidade a um fenômeno doloroso agudo.^16^

Os instrumentos identificados foram apresentados nas tabelas 1 e 2 conforme classificação em unidimensionais e multidimensionais, bem como quanto a tipo de dor aguda e prolongada.

**Tabela 1:** Distribuição dos instrumentos segundo a classificação unidimensional e os tipos de dor. Fortaleza, CE, Brasil, 2012

**Tabela 2:** Distribuição dos instrumentos segundo a classificação multidimensional e os tipos de dor. Fortaleza, CE, Brasil, 2012

A maioria dos instrumentos é unidimensional, para dor aguda, e utiliza alguns dos indicadores comportamentais: expressão facial, choro e atividade motora. No caso da ABC *pain scale*, por exemplo, avalia a dor por meio de características do choro do RN: tonalidade do primeiro choro, ritmo dos acessos de choro e constância da intensidade do choro. Enquanto que a *Neonatal Pain Analyzer* - ABC *analyzer* se baseia, além da tonalidade do choro, em outros aspectos do mesmo indicador, como: raiz quadrada média (RMS) da amplitude normalizada do choro e presença de choro em sirene.^17^^-^ 19

O choro é a forma de comunicação mais primitiva, sendo considerado, simultaneamente, um sinal, um sintoma e um indicador. O significado do choro ainda não está claro, visto que suas diferentes características podem remeter a diferentes níveis de estresse, relacionados a diversas causas, tal como dor, fome ou desconforto.^20^^-^ 21

Ainda para dor aguda, destacam-se DAN, EDIN e BIIP. Além do indicador comportamental relacionado à expressão facial, também utilizaram outros aspectos comportamentais, a saber: a DAN com a expressão facial, movimentos dos membros e expressão vocal. A EDIN por meio de expressão facial, movimentos corporais, qualidade do sono, qualidade de interação, reconforto/consolabilidade e a BIIP com o estado sono/vigília, cinco diferentes trejeitos faciais e dois diferentes movimentos das mãos.^17^^,^ 22 ^-^ 23

Das unidimensionais para dor prolongada, a VAS avalia apenas a expressão facial^24^ e a LIDS, os movimentos corporais, excitabilidade, flexão dos dedos da mão e do primeiro dedo do pé, tônus, expressão facial (quantidade e qualidade) e sono. A CHIPPS, a RIPS e a NAPI associaram os três indicadores comportamentais: expressão facial, choro e atividade motora, além de outras, como resposta ao toque na NAPI, consolabilidade e resposta ao toque.^17^^,^ 25 ^-^ 27

A DSVNI utilizou indicadores comportamentais, baseados em cinco escalas - NFCS, The *Infant Body Coding System* (IBCS), *Neonatal Behavioural Assessment Scale, Assessment of Preterm Infant's Behaviour* e *Gustave Roussy Child Pain Scale*, mas que não foram divulgados no estudo selecionado, pois esta escala não foi publicada e não está sendo usada na prática clínica.^17^^,^ 28

A NFCS e a FLACC avaliam dor aguda e prolongada. A NFCS utiliza apenas aspectos da expressão facial como testa e olhos franzidos, aprofundamento do sulco nasolabial e estiramento horizontal da boca. Enquanto que a FLACC mensura com todos os tipos de indicadores comportamentais: expressão facial, movimentos dos membros inferiores, atividade corporal choro e dificuldade em consolar.^17^^,^ 29 ^-^ 31

Nos RN e bebês, a dor tende a manifestar-se por meio do choro e dos movimentos corporais, expressões faciais ou mesmo apatia. Em crianças com idades compreendidas entre um e três anos, o choro pode surgir acompanhado por verbalizações ou gestos localizados na região do foco da dor, e por determinados movimentos inquietos, violentos ou de birra.^32^

Sendo o choro, expressões faciais e atividade motora indicadores observados em outras situações do RN, como estresse e desconforto, por exemplo, alguns pesquisadores buscaram desenvolver escalas que associaram indicadores comportamentais e fisiológicos para obter uma avaliação mais acurada da dor.

As expressões multidimensionais de dor indicam que a avaliação não deve focar apenas os sinais comportamentais selecionados, mas capturar todas as expressões possíveis da dor.^33^ Respostas à dor também são influenciadas por tipo de estímulo, estado de sono-vigília, idade de desenvolvimento, a gravidade da doença, o uso de agentes farmacológicos e sua quantidade, tipo e tempo de exposições de dor.^34^

Dos instrumentos multidimensionais para dor aguda, destaca-se a PAIN com indicadores como: expressão facial, choro, padrão respiratório, movimento das extremidades, estado de alerta, saturação de oxigênio e frequência cardíaca;^35^ a IBCS por meio da expressão facial, com movimentos corporais, características do choro, frequência cardíaca;^36^ a BPSN utiliza o estado de alerta, duração do choro, tempo para acalmar, cor da pele, expressão facial, postura, padrão respiratório, frequência cardíaca e saturação de oxigênio.^37^

A SUN possui indicadores de frequência cardíaca, respiração, pressão arterial média, estado de alerta, movimento, tônus, expressão facial.^38^ A NIPS com expressão facial, choro, padrão respiratório, movimentos dos membros superiores, movimentos dos membros inferiores e estado de alerta^17^^,^ 25 e a Hartwing avalia a resposta motora, expressão facial, abertura ocular, frequência respiratória e reação à aspiração orotraqueal.^39^

A PASPI utiliza transição entre os estados de sono, expressões faciais, mudanças na frequência cardíaca e saturação de oxigênio, a movimentos do corpo e membros e comportamentos das mãos.^40^ A NNICUPAT traba-lha por meio da expressão facial, movimentos corporais, cor da pele; saturação de oxigênio; frequência respiratória, frequência cardíaca e percepção da dor pelo(a) enfermeiro(a).^17^^,^ 41

Para avaliação da dor prolongada, apresenta-se a CRIES, que trabalha com indicadores como o choro, expressão facial, saturação de oxigênio, sinais vitais e padrão do sono.^25^ A N-PASS, com o choro/irritabilidade, estado/comportamento, expressão facial, tônus/extremidades, sinais vitais (frequência cardíaca, frequência respiratória, pressão arterial e/ou saturação de oxigênio).^42^ A MAPS, por meio da frequência cardíaca e pressão arterial, padrão de respiração, expressão facial, movimentos corporais e estado de alerta^43^ e a PAT avalia expressões faciais, choro, postura do corpo, sono, percepção da enfermeira, coloração, frequência cardíaca, padrão respiratório, pressão arterial e saturação de oxigênio.^17^^,^ 44 ^-^ 45

A COMFORT *scale* é um instrumento de avaliação de dor multidimensional que possui indicadores comportamentais: alerta, agitação, reação respiratória, choro, movimentos gerais, o tônus muscular e expressão facial, e os fisiológicos: pressão arterial e a frequência cardíaca. Essa escala foi especialmente desenvolvida para avaliar medidas de sofrimento causado por dor em crianças de 0 a 18 anos internadas em Unidade de Terapia Intensiva (UTI).^17^^,^ 46 ^-^ 47

A *Adapted *COMFORT *Scale* é proveniente da COMFORT *scale* que foi submetida a uma pesquisa de validação com recém-nascidos prematuros com menos de 35 semanas de idade gestacional, utilizando todos os itens da escala original, exceto a avaliação da pressão arterial invasiva.^17^^,^ 46 ^-^ 47 A COMFORT-B foi derivada da escala COMFORT original, excluindo os dois parâmetros fisiológicos (frequência cardíaca e pressão arterial média sanguínea), mantendo apenas indicadores comportamentais: alerta, agitação, reação respiratória, choro, movimentos gerais, tônus muscular e expressão facial. No entanto, o aspecto da reação respiratória, considerado um componente fisiológico, mantém essa escala como multidimensional.^17^^,^ 46 ^-^ 48

A escala PIPP avalia a idade gestacional, estado de alerta, frequência cardíaca, saturação de oxigênio e expressão facial (testa franzida, olhos cerrados, aprofundamento do sulco nasolabial). É a única escala multidimensional que, entre os seus indicadores, inclui a idade gestacional para avaliar a dor do RN termo e RNPT.^17^^,^ 49

Quanto a faixa etária por escala, observou-se que a CHIPPS e a COMFORT-B avaliam a dor em crianças com idade de zero a 5 anos; a FLACC, RIPS, CONFORT e a NAPI, de zero a 3 anos; a VAS, de zero a 4 anos; a MAPS, de zero a 31 meses e a Hartwing de zero a 1 ano,^17^^,^ 39 ^,^ 44 ^,^ 50 as quais são comuns em RN e crianças.

No que se refere ao estímulo doloroso, observou-se o uso na punção do calcâneo (*ABC pain scale, Neonatal Pain Analyzer - ABC analyzer*, DAN, IBCS, PIPP, NIPS, PASPI), na venopunção (DAN, BIPP, PIPP, NIPS), na ventilação mecânica (EDIN, N-PAN, NNICUPAT), na aspiração traqueal (PAIN, COMFORT), após cirurgia (EDIN, CHIPPS, FLACC, LIDS, NAPI, RIPS, VAS, NFCS, N-PAN, PIPP, PAT, MAPS, COMFORT, COMFORT *Scale*), no procedimento doloroso de rotina (DSVNI) e na queimadura (VAS).^17^^,^ 44

 Relacionando os instrumentos ao contexto de aplicação, percebeu-se que a escala VAS foi avaliada em pacientes com artrite reumatoide,^48^ a Hartwig em RN sob ventilação mecânica durante a aspiração traqueal,^39^ a COMFORT-B em crianças com síndrome de Down internadas em UTI^48^ e a BPSN em RN com ou sem ventilação por pressão positiva.^37^. Ressalta-se que uma mesma escala pode ser utilizada para avaliar a dor em diferentes contextos.^51^

Em relação às propriedades psicométricas das escalas de dor, a validade de critério foi predominante nos estudos selecionados. O PASPI^40^ e o COMFORT-B foram os únicos instrumentos que passaram pela validade de conteúdo, critério^47^^,^ 48 e construto.^48^^,^ 50

Pelos dados da pesquisa de revisão sistemática, a VAS foi comparada com *Modified Infant Pain Scale* (MIPS) e verificou-se elevado grau de concordância ao classificarem os RN como confortáveis ou não confortáveis após cirurgia eletiva. Constatou-se, também, que essa escala foi usada para validar a NIPS, CONFORT e a NNICUPAT. Junto com a CONFORT para validar a NFCS, e com a PIPP, para validar a BPSN, e também com a FLACC, para validar a MAPS em crianças dos 0-31 meses.^17^ Portanto, a VAS foi uma das escalas mais utilizadas para validação de critério. 

Ainda sobre a validação de critério, a COMFORT foi usada em RN com a VAS para validar a NFCS. A confiabilidade da escala foi dada pelo Kappa (0.62-0.84) e pelo coeficiente intraclasse (0.85).^46^ A escala COMFORT-B mostrou adequada consistência interna quando comparado com a Escala de Avaliação Numérica (NRS) em crianças com síndrome de Down, com valor alfa de Cronbach (0.84-0.87).^48^

A confiabilidade interobservador de NFCS foi avaliada em vários estudos, tanto na versão modificada da escala com quatro medidas quanto na convencional com dez medidas. A versão da escala com dez medidas obteve um valor de 0.89, com uma amostra mista de RN termo e RNPT e a amostra com quatro medidas obteve uma confiabilidade interobservador de 0.91.^17^ ^,52.^

Em revisão para avaliar as propriedades de medida e estudos de intervenção com a PIPP, verificou-se que o instrumento continua a ser uma medida confiável e válida para avaliar a dor aguda em crianças. A confiabilidade interclasse foi excelente (>0.89) e a confiabilidade intra-examinadores foi de 0.95.^49^ Outro estudo de revisão, com a mesma escala de dor em RN termo e RNPT, obteve uma excelente confiabilidade interobservador, de 0.93 a 0.96, e intraobservador, de 0.94 a 0.98.^17^

## Conclusão

O presente estudo mostrou que existem pelo menos 29 escalas de avaliação de dor em RN disponíveis em publicações científicas da área de neonatologia, sendo 13 unidimensionais e 16 multidimensionais, que contemplam RNPT e RN a termo, em situações de dor aguda e prolongada.

Os artigos selecionados apresentaram nível V de evidência, ou seja, evidências originárias de revisão sistemática de estudos descritivos ou quantitativos, em que a maioria foi estudos do tipo metodológico de validação, convergindo com os objetivos do presente estudo.

Com base no conhecimento das características de cada escala, não se pode eleger a mais adequada, pois a esco-lha dependerá da idade gestacional, do tipo de estímulo doloroso, do contexto em que o RN se apresente. Por isso, ressalta-se que, diante dos estudos, ainda não existe uma escala padrão ouro para avaliação da dor no RN. O profissional deve utilizar escalas validadas, confiáveis, seguras e práticas à beira do leito, podendo ser uni ou multidimensionais, principalmente devido às divergências existentes na literatura.

Frisa-se que a avaliação da dor no período neonatal deve ser multiprofissional, devido à subjetividade do fenômeno avaliado e das escalas disponíveis, quanto mais profissionais das diversas áreas da saúde avaliarem o mesmo RN utilizando escalas diferentes, talvez possa-se aumentar a objetividade desta avaliação. 

Destaca-se também a necessidade imperiosa dos serviços possuírem, utilizarem e atualizarem as rotinas e protocolos para a avaliação e tratamento da dor no RN, assim como o treinamento e capacitação dos profissionais que trabalham nestas unidades, garantindo a aplicação na prática dos conhecimentos relacionados à prevenção, avaliação e tratamento da dor, com objetivo de uniformizar a atuação dos profissionais do serviço e permitir um tratamento adequado aos RN.

Especificamente em relação às escalas de avaliação de dor, é importante que, antes da administração o profissional de saúde conheça os detalhes das dimensões avaliadas, a operacionalização de uso e os aparelhos necessários para avaliação condizentes com a proposta do instrumento.

Reconhece-se a necessidade da utilização de métodos de avaliação de dor mais específicos e acurados em neonatologia, devido à subjetividade da dor, principalmente em uma população que não verbaliza a sensação dolorosa. Assim, almeja-se que, por meio deste estudo, os profissionais da equipe multiprofissional possam escolher a escala de avaliação de dor mais adequada para seu campo de atuação, disponibilidade de tempo, população, tipo de dor e validade.
